# Prevalence of Low Self-Esteem Among Patients Treated in the Craniofacial Surgery Department of an international Reference Hospital

**DOI:** 10.1192/j.eurpsy.2024.1006

**Published:** 2024-08-27

**Authors:** G. Pereira Bernd, V. Dall Agnol Bouvier, T. Brusa da Costa Linn, I. Cho de Almeida, B. de Oliveira de Marchi, L. Guinter Muccillo, C. G. Menezes Chaves Barcellos, C. Paz Portinho, M. V. Martins Collares, R. Ribas Leite

**Affiliations:** ^1^Universidade Federal do Rio Grande do Sul, Porto Alegre; ^2^Feevale, Novo Hamburgo; ^3^Pontifícia Universidade Católica do Rio Grande do Sul, Porto Alegre, Brazil

## Abstract

**Introduction:**

Craniofacial surgery is a specialized field that addresses congenital and acquired deformities of the head and face. While the physical outcomes of craniofacial surgery are well-documented, less attention has been given to the psychological well-being of adult patients. This abstract aims to explore self-esteem issues among adult patients treated at the Craniofacial Surgery Sector of HCPA (Hospital de Clínicas de Porto Alegre), where a substantial proportion of adult patients have reported self-esteem problems.

**Objectives:**

1. To assess the prevalence of self-esteem issues among adult patients (≥18 years old) attending the HCPA Craniofacial Surgery Sector.
2. To examine potential contributing factors to self-esteem problems in this specific patient population.
3. To evaluate the impact of self-esteem on the mental health and psychosocial functioning of adult craniofacial surgery patients.
4. To propose recommendations for psychosocial support and intervention strategies tailored to the needs of adult patients in this context.

**Methods:**

This cross-sectional study involved 132 adult patients who had undergone or were scheduled for craniofacial surgery at HCPA. Participants reported self-esteem issues in their talk with the hospital’s physicians, and their medical records were reviewed to collect demographic and clinical data. Additionally, participants provided information about their mental health status and psychosocial functioning.

**Results:**

Among the 39 adult patients included in the study, 37 (94.9%) reported experiencing self-esteem issues, such as lack of confidence or feeling unattractive. The most commonly reported contributing factors were visible facial differences, social interactions, and prior surgical experiences. Patients with lower self-esteem had a higher likelihood of reporting symptoms of depression and anxiety and reported lower overall psychosocial functioning compared to those with higher self-esteem.

**Conclusions:**

This reveals a strikingly high prevalence of self-esteem issues among adult patients attending the Craniofacial Surgery Sector at HCPA. These findings underscore the importance of recognizing and addressing the psychological well-being of adult craniofacial surgery patients. Comprehensive psychosocial support, including counseling, peer support, and interventions to enhance self-esteem, should be integrated into the care of these patients. By addressing self-esteem concerns, healthcare providers can improve the mental health and overall quality of life of adult craniofacial surgery patients.

**Disclosure of Interest:**

None Declared

